# Age-Friendly Health Care in Rural Washington: Assessing System Readiness and Advancing Recognition

**DOI:** 10.1093/geroni/igaf122.3878

**Published:** 2025-12-31

**Authors:** Anita Souza, Katherine Bennett, Aimee Verrall, Leigh Ann Mike, Barbara Cochrane, Elizabeth Phelan

**Affiliations:** University of Washington, Seattle, Washington, United States; University of Washington School of Medicine, Seattle, Washington, United States; University of Washington / School of Medicine, Seattle, Washington, United States; University of Washington, Seattle, Washington, United States; University of Washington, Seattle, Washington, United States; University of Washington, School of Medicine, Seattle, Washington, United States

## Abstract

Aging demographic trends in Washington State reflect national patterns, with rural communities in particular experiencing significant growth in adults over age 65 along with an overwhelmed healthcare workforce and accessibility challenges. As this population expands, health systems are called upon to collaborate with older adults, address their unique health needs, and apply the 4Ms of Age-Friendly Health Systems across the continuum of care. To support this mandate, this project engaged rural health systems to formalize Age-Friendly care by implementing structured assessments and evidence-based improvements for Institute for Healthcare Improvement (IHI) recognition. An Age-Friendly Outreach Coordinator provided targeted support and strategic collaboration with three health systems in rural counties. Each system reviewed its current care and then implemented education and evaluation to align with Age-Friendly care across its sites. Within six months, all three participating health systems – including five primary care clinics, one critical access hospital, and one nursing home – achieved IHI’s Age-Friendly Level 1 recognition. This consistent attainment of Level 1 highlights the feasibility and adaptability of Age-Friendly interventions in different rural sites and demonstrates the readiness of rural providers to support older adults through integrated, person-centered models of care. These findings suggest that with targeted support and strategic collaboration, Age-Friendly practice can be promoted in rural healthcare organizations, with the goal of improving outcomes and care experiences for older adults. Future efforts will focus on sustaining these changes, technical assistance to track Age-Friendly care processes and outcomes and expanding the evaluation/dissemination of outreach coordinator support.

